# Report of a Case of Supposed Hysterical Convulsions

**Published:** 1854-09

**Authors:** 


In giving the case recorded below, our readers will notice the nomenclature assigned to the disease. Hysteria, though etymologically inapplicable to "a stout negro lad," is, undoubtedly, the best name Dr. Little could have bestowed upon the case. A local irritation of a mucous membrane, reflected through the ganglionic upon the cerebro-spinal system, and causing convulsion, expresses at once the mode of action of hysteria, of infantile convulsion from teething or intestinal irritation, and of cases like that described.

*Report of a Case of Supposed Hysterical Convulsions.* By JOHN P. LITTLE, M. D., of Richmond.

I offer this case under the above title, with some doubt as to whether the disease has been rightly named, or whether the term hysteria can justly belong to the symptoms which I will describe. The attack appeared to be caused by an irritating substance passing through the intestinal canal. And it may be recollected by the readers of the Stethoscope, that a short time ago Dr. Dove reported a case where a large and ragged piece of bone passed through this canal, causing no disturbance whatever until it reached the rectum. My own case was exactly the reverse of this one: the substance was not hard or ragged, (being a large piece of gristle,) it passed readily enough from the rectum when that outlet was reached, and it was in the passage through the bowels ehat the convulsive paroxysms occurred, which made the case interesting by their peculiar character.

September 11, 1853—A stout negro lad, belonging to Mr. Braxton near Richmond, had eaten the night before a part of a beef's head, and had swallowed the eye almost unchewed. Early on the next morning he was seized while on his way to market with severe pain in the abdomen, followed by convulsions. He became for a few minutes as rigid as a piece of wood. The case was pronounced tetanic, and calomel and opium were prescribed by same neighboring physician. I saw him in the evening and found him laboring under fever, complaining of pain in the head and abdomen; the latter was tender on pressure. There had been no return of the convulsions, and the boy was perfectly conscious. Attributing the attack to an inordinate meal of indigestible food, I prescribed an active cathartic and left him. By daybreak I was hastily summoned to him, and learned that he had slept quietly until 2 o'clock, and that he had had twenty "fits" since that time. He was free from pain, perfectly conscious, without fever, and with a pulse of 65; yet, although his medicine had acted well, his abdomen was still tumid and somewhat tender on pressure.

In a few minutes he was seized with one of the so called fits. He cried out that it was coming, complained of severe pain in the belly, and writhed as if suffering; in an instant he passed into a state of unconsciousness, the trunk bent back as in opisthotonos, the limbs moving, yet not convulsed, the lips slightly covered with foam, and the abdomen tumid and hard, yet there was no rigidity except in the abdominal muscles; there was no convulsion, no stertorious breathing, nor was the countenance distorted. The duration of the attack was not longer than a moment, and he recovered from it both speedily and entirely. These curious attacks continued during the day; sometimes they would occur every ten minutes, sometimes there was half an hours interval between them, sometimes only one occurred, and then again three, four or five followed each other in rapid succession. They were not increased by moving him. After the occurrence of a succession of these paroxysms he would sleep heavily for a short time; yet there was no coma, for he could be easily roused. Indeed, there was no drowsiness, no headache or uneasiness about the head. He remained awake and free from any pain, even in the abdomen, in the intervals between the paroxysms. There was no nausea; he drank water freely and retained it. I administered active purgatives and used injections: they acted powerfully, and the paroxysms were increased in frequency by the purgation. Fomentations to the abdomen, and opium by the mouth and by the rectum were employed without any permanently good effect.

After using these various remedies from 5 o'clock A. M. until 2 o'clock P. M., it was plain that the symptoms were caused by an irritation that had its seat in the nervous system; and as the brain and the spinal system were not primarily affected; as, moreover, the abdomen was pointed out, both by the history of the case and the character of the attack, as the seat of the disease, I became convinced that an irritation transmitted from the lining membrane of the intestines to the ganglionic or sympathetic nerves, and through them to the cerebro-spinal system, was the cause of these singular attacks. Supposing this excitement or irritation to be analogous to that of hysteria, I determined to use the sedative or antispasmodic so much relied on in that affection. I gave a dose of the mist. assafœtida of the Dispensary, and placed a large blister over the entire abdomen. This blister did not draw at all, being badly made; the assafœtida acted like a charm; he had no paroxysm for three hours. The mixture was ordered to be continued every hour, and was so used for twenty-four hours. During this time he had four paroxysms, at long intervals and slight in character; at the end of which time he passed the gristly substance which had caused all this trouble. The attacks now ceased entirely, and the boy recovered rapidly.

I have ventured to call this case an hysterical one, because relief was afforded by *the* remedy of that complaint. I am sensible that I have laid myself open to criticism thereby, yet I do not know how else to name the disease. It is true, that the resemblance to hysteria is by no means perfect. The patient was a stout negro lad. The cause of the attack was plainly due to the swallowing of an indigestible substance. There was no laughing, sobbing, or globus hysterius, nor was there any violent convulsion, such as often occurs in hysteria. The seat of the affection, and the source from which the symptoms had their rise, was evidently the ganglionic system, that large nervous apparatus which presides over organic life, and of which hysteria is but a diseased and disordered function.

Had this patient been a woman, and especially a delicate woman, I doubt not that the case would have been one of decided hysteria. If it had been a child, the brain and the spinal system would have been more affected.

I regret that I did not make use of chloroform during the violence of these attacks. Administered by the mouth or through the rectum, it would probably have given me an earlier control of the disease.—*Ibid.*

